# *In Silico* Evaluation of Bioactive Compounds from Cucumis anguria L. as Potential Inhibitors of Antibiotic-Resistant New Delhi Metallo-β-Lactamase (NDM-1)

**DOI:** 10.1021/acsomega.5c02627

**Published:** 2025-05-30

**Authors:** Soundar Rajan Kulandhaivel, Pandiyan Muthuramalingam, Balasubramanian Sivaprakasam, Manikandan Ramesh, Arun Muthukrishnan, Kapildev Gnanajothi, Vidhyavathi Ramasamy, Thamaraiselvi Chandran, Hyunsuk Shin, Jesudass Joseph Sahayarayan

**Affiliations:** † Department of Bioinformatics, Science Campus, 29942 Alagappa University, Karaikudi 630003, Tamil Nadu, India; ‡ Division of Horticultural Science, College of Agriculture and Life Sciences, 26720 Gyeongsang National University, Jinju 52725, South Korea; § Department of Biotechnology, Science Campus, Alagappa University, Karaikudi 630003, Tamil Nadu, India; ∥ Department of Biotechnology, 29897 Bharathiar University, Coimbatore 641 046, Tamil Nadu, India; ⊥ Translational Plant Research Laboratory, Department of Microbial Biotechnology, Bharathiar University, 641 046 Coimbatore, Tamil Nadu, India; # Department of Biotechnology, Mother Teresa Women’s University, Kodaikanal 624101, Tamil Nadu, India

## Abstract

Traveler’s diarrhea (TD), manifested by loose stools, is a critical health issue affecting the digestive system. It poses a significant health risk with documented mortality and morbidity. Escherichia coli and *Klebsiella* are primary causative organisms. The emergence of New Delhi metallo-β-lactamases (NDM-1), initially reported in E. coli and Klebsiella pneumoniae, has driven the rapid dissemination of antibiotic-resistant strains. The *bla NDM-1* gene encodes NDM-1, an enzyme that confers resistance to β-lactam antibiotics. Traditionally, Cucumis anguria L., which is native to Africa and is widely distributed in East and Southern Africa, has been used to alleviate various stomach disorders. The NDM-1 metallo-β-lactamase protein is involved in the production of β-lactams. In this study, we have conducted *in silico* screening of natural bioactive compounds, including ethylenediaminetetraacetic acid (EDTA), to identify potential inhibitors of metallo-β-lactamase protein. Molecular docking is performed to evaluate the binding interactions between the compounds and NDM-1. Subsequently, the ADME/Tox properties of the lead compounds and EDTA are predicted. The phytocompounds homogentisic acid, caffeic acid, and protocatechuic acid have Glide g-scores of −8.818, −8.663, and −8.121 kcal/mol, respectively. They form hydrogen bonds with GLN 123 and ASN 220, along with metal coordination involving Zn^2+^ ions. Molecular dynamics (MD) simulations, including RMSD, RMSF, *R*_g_, and hydrogen bond analyses, are conducted on the top-ranked protein–ligand complexes and EDTA. The results indicate that the protein–ligand complexes remained stable throughout the 100 ns simulation period. This assessment provides the first evidence supporting the specificity and compatibility of potential phytochemicals in C. anguria against TD by inhibiting β-lactam proteins.

## Graphical abstract

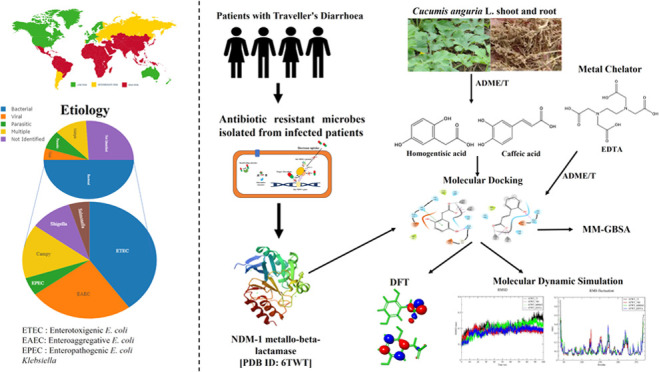

## 1 Introduction

Traveler’s diarrhea (TD) is an intestinal infection characterized by loose stools and abdominal cramps, commonly resulting from the consumption of contaminated food or water. Travelers visiting regions with climates and sanitation standards that differ from those of their hometowns face a higher risk of illness. This risk often arises from inadequate sanitation practices among food handlers or the consumption of contaminated food.^1,2^ The most frequent cause of TD is believed to be bacterial infection, accounting for 80–90% of cases. According to the area, studies suggest that between 9 and 79% of people traveling to Eastern and Southeastern Asia suffered from TD during or after their trip.^3^ These incidences are high in warmer climates, where sanitation and hygiene practices are deplorable. Various antibiotics such as loperamide, rifaximin, and fluoroquinolones are used to treat TD. Sometimes, β-lactamase resists all of the antibiotics.^4^ Thus, multidrug resistance mechanisms emancipated by TD account for critical health hazards. Every year, around 80 million tourists traveling abroad experience diarrhea. As a result, TD will remain a global concern.^5^

TD is mainly caused by the New Delhi β-lactamase (NDM-1) protein, which inactivates all β-lactams, except aztreonam.^6,7^ The gene renders bacteria resistant to antibiotics of the carbapenem family. NDM-1 was also reported in a Swedish patient of Indian origin in 2008, incited by Escherichia coli, although initial isolation correlates to Klebsiella pneumoniae.^8^ Subsequently, the prevalence was confirmed in the UK, the US, Canada, Japan, India, and Pakistan. The *bla NDM-1* gene encodes NDM-1 protein, a carbapenemase β-lactamase enzyme that hydrolyzes and inactivates carbapenem antibiotics.^9^ The first documented fatality caused by bacteria expressing the NDM-1 enzyme occurred in August 2010. A significant quantity of the *bla NDM-1* gene was isolated from microbes found in tap water and sewage water collected from hospitals and urban areas.^10,11^

NDM-1 has recently been reported to be resistant to carbapenemases, spreads rapidly, and has major consequences in the field of clinical microbiology.^12,13^ Different types of NDM-1 inhibitors have been tested against microbes. Thus, the presence of an infection carrying the NDM-1 gene poses a serious threat and consequently may lead to mortality and fatal sequel.^14^ Creating novel β-lactam molecules that are resistant to hydrolysis by these enzymes and the development of β-lactamase inhibitors, which can be combined with antibiotics to create a single product because β-lactamase inhibitors extend the shelf life of outdated β-lactam antibiotics, are the two major strategies used to neutralize β-lactamases. Clinically approved metallo-β-lactamase inhibitors are still unknown.^15^ Based on the CDC 2024 report, there are no available vaccines or medicines to cure TD. However, antibiotics such as Azithromycin, Ciprofloxacin, Levofloxacin, and Rifamycin have been used to reduce the risk of illness. NDM-1 poses a significant public health threat by hydrolyzing a broad range of β-lactam antibiotics because of the absence of clinically approved inhibitors available to neutralize its zinc-dependent activity, leading to treatment failures and high mortality rates.^16^ Targeting NDM-1 with novel zinc-chelating agents from natural resources offers a promising strategy to restore the effectiveness of β-lactam antibiotics without side effects and combat the growing challenge of antimicrobial resistance.

Structural conformations and changes are deciphered as serine or zinc ions that are present at the dynamic centers of β-lactamases and have electrostatic interactions with carbapenemase enzymes, hence called metallo-β-lactamases (MBLs). Most of the MBLs have two zinc metal ions, in which Zn1 can bind to the molecule through hydroxyl groups and Zn2 with the substrate and is mediated by a metal coordination bond and Zn2.^17^ The MBLs can be categorized into three subclasses, such as B1, B2, and B3, according to sequence similarity.^18^ The metal chelators, like ethylenediaminetetraacetic acid (EDTA), are used to inhibit NDM-1 by sequestering essential zinc ions from its active site.^19,20^

Mutations in NDM-1 can significantly influence protein stability, catalytic efficiency, and substrate specificity, impacting their role in antibiotic resistance.^21^ Some mutations can enhance the thermodynamic or structural stability of NDM-1, allowing it to function more effectively in diverse bacterial hosts or under varying environmental conditions. Mutations near the active site, especially those involving residues that coordinate with zinc ions or interact directly with β-lactam antibiotics, can alter the enzyme’s hydrolytic activity. Natural mutations in NDM-1 variants (like NDM-4, NDM-5, etc.) have shown increased resistance to the antibiotics.^20^

Cucumis anguria L., commonly known as bur gherkin, is an underutilized traditional medicinal plant that belongs to the Cucurbitaceae family and genus *Cucumis*. This type of plant produces bitter fruits and nonbitter fruits.^22^ The leaves and fruits of this bur gherkin are consumed as pickles and used as fresh fruits in salads. Every part of this plant belongs to the realm of traditional medicine, combating stomach-related problems and kidney stones.^23,24^ The fruit from this plant contains large amounts of protein, iron, potassium, phosphorus, calcium, and vitamin C. Further, the plant is mainly consumed and cultivated in many countries like the United States, Brazil, India, Cuba, Africa, and Zimbabwe.^25^ Phytochemicals from C. anguria are used to treat jaundice, hemorrhoids, stomach aches, and kidney stones. The reported phytochemicals include Cucurbitacin B, Cucurbitacin D, and Cucurbitacin G, where Cucurbitacin B is used to prevent cancer. Compounds from C. anguria L. have high levels of antioxidant activity.^26^ Japanese gherkins displayed a low concentration of carotenoids, nondetectable amounts of anthocyanins, and vitamin C, and a moderate source of phenolic compounds. The antibacterial properties of C. anguria leaf extract were studied and found to act as a potent antibacterial agent, notably against K. pneumoniae, S. aureus, and E. coli.^27^

Here, we chose the N-terminally truncated NDM-1 metallo-beta lactamase [PDB ID: 6TWT] crystal structure for docking studies. In this structure, two positively charged zinc metal ions are present at the active site and are responsible for the hydrolysis of the β-lactam ring in NDM-1, a major factor contributing to antibiotic resistance in certain bacteria.^28^ This protein corresponds to the B1 subclass (broad-spectrum enzyme), and coordination spheres of zinc ions are present at the active center of B1 with water molecules.^29^ Therefore, developing selective metal chelators from the phytocompounds from C. anguria that specifically target the zinc ions in NDM-1 without affecting other metalloproteins is a promising strategy to combat antibiotic resistance mediated by this enzyme.

## 2 Methodology

The workflow combines computational methodologies to systematically identify and characterize potential drug-like compounds, as depicted in Figure 1. By integrating ligand-based screening, docking, molecular dynamics, and quantum chemical calculations, this approach facilitates the identification of promising phytocompounds from C. anguria for *NDM-1* inhibition.

**Figure 1:**
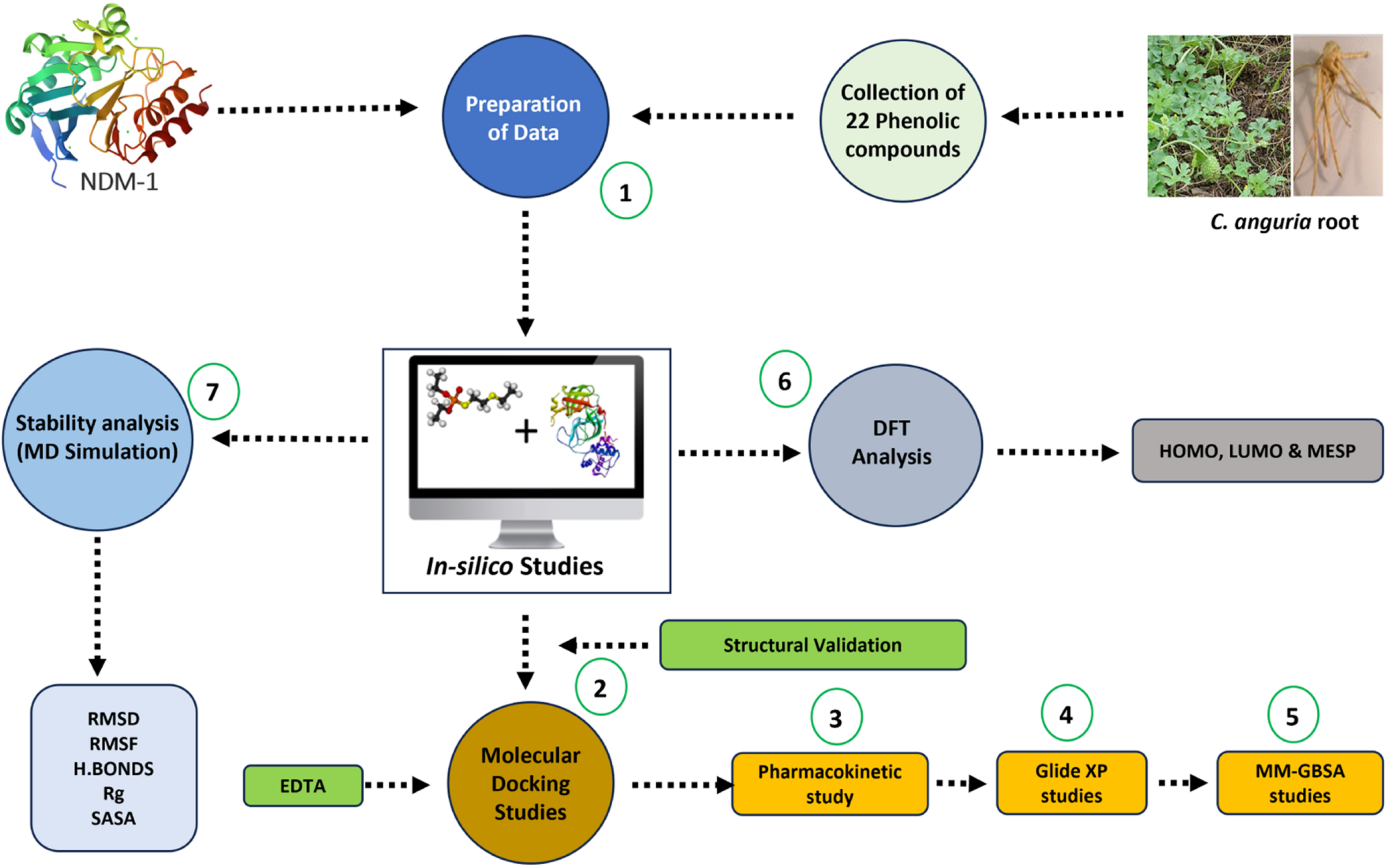
Ligand-based virtual screening workflow implemented in this study. The workflow includes the identification of active phytocompounds, molecular docking, ADMET prediction, and molecular dynamics simulations.

### 2.1 Data Set

Phenolic compounds from the roots of C. anguria were collected through a literature survey,^30^ and compound structures were retrieved from the PubChem database. The retrieved compounds were optimized with the LigPrep module from Schrodinger and subjected to further analysis. The crystal structure of *bla NDM-1* (PDB ID: 6TWT) with reasonable resolution was retrieved from the PDB database for screening studies with the GLIDE (GLIDE, Maestro, Schrodinger 2023-2) docking protocol. Water molecules were removed from the crystal structure and preprepared by the Protein Preparation Wizard in Schrodinger. The OPLS-2005 force field was employed to minimize energy. This study mainly focused on identifying potent compounds from the roots of C. anguria that can inhibit the *bla NDM-1* receptor.^31^

### 2.2 PROCHECK Analysis

The geometrical parameters of the protein were evaluated using PROCHECK, which can validate the quality of protein models by assessing the dihedral angles (phi and psi), bond lengths, and bond angles. This process ensures the accuracy and reliability of the protein structure for further study.

### 2.3 Drug Likeness and ADME/Tox Prediction: Computational Approach

Compounds with unconditional pharmacokinetic properties like absorption, distribution, metabolism, and excretion (ADME) have been considered unsuitable for drug development.^32^ The selected compounds from the plant C. anguria root were subjected to ADME properties by using the Qikprop version to decipher the drug likeness (QPlog Po/w, QPlogS, QPlog HERG, QPlog BB levels) and human oral absorption percentage and rule of 5 (RO5) properties aiding the drug discovery process. ProTox-III (https://tox.charite.de/protox3/index.php?site=compound_input) is an online web server that predicts the toxicity of small molecules.^33^

### 2.4 Molecular Docking: Ligand-Based Screening

The sitemap module was used to identify the substrate-binding pocket of the *bla NDM-1* receptor. A comprehensive structural analysis revealed the remarkable conservation of the binding pocket, suggesting a promising possibility for developing potential inhibitors targeting *bla NDM-1*. Compounds derived from medicinal plants that inhibit NDM-1 activity are considered promising candidates for traditional drug design. In this screening, the selected compounds underwent a sequential docking process, including ligand docking and extra precision (XP) modes with default parameters to retain the best-scoring conformations. Docking calculations were conducted by using the OPLS-2005 force field. Following a successful screening analysis, the top compounds were selected based on predefined inclusion criteria.^34^

### 2.5 Free Binding Energy Analysis: Prime MM-GBSA

To evaluate the relative free energy of ligand binding at the protein’s active site, the top molecules identified through Glide docking were analyzed. The prime MM-GBSA module was used for this analysis. This approach utilizes the OPLS-2005 force field to compute the energies of the complex. The equation used to calculate the binding energy is as follows

$$\Delta G_{\mathrm{binding}}=\Delta G_{\mathrm{complex}}-\left[\Delta G_{\mathrm{protein}}+\Delta G_{\mathrm{ligand}}\right]$$

where Δ*G*_binding_ is the free energy of the protein–ligand complex, and Δ*G*_protein_ and Δ*G*_ligand_ are minimized energy values of the protein and ligand, respectively.^35^

### 2.6 Density Functional Theory (DFT) Analysis

DFT calculations were applied to the top-scoring compounds, and EDTA was obtained from molecular docking and ADMET studies. The Jaguar module, Maestro, Schrodinger 2023-2, was used to calculate various electrostatic properties such as HOMO, LUMO, dipole moment, electron density, and electrostatic potential of the lead molecules. DFT is also useful to get detailed geometric features of the molecule.^36^

The lead compounds were optimized using the Lee–Yang–Parr correlation functional (B3LYP) theory with the 6-31G**++ basis set to determine the compound-binding capabilities more effectively. The water solvation system, Poisson–Boltzmann finite, dispenses the molecules. The electron donor and acceptor region of the molecules were identified by HOMO and LUMO, and their energy gaps well defined the stability and bioactive properties of the lead molecules.^37^ The charge distribution of the molecule, indicated by its van der Waals contact area, was measured by electrostatic potential energy represented by the colored surface of the molecule. The positive potential regions of the molecule were colored deep blue, while the negative regions were deep red. The electronegativity, hardness, and softness of the compounds were determined as follows^38^

(a) The energy gap is the difference between the energies of the LUMO and the HOMO:
$$\mathrm{energy}\;\mathrm{gap}=E_{\mathrm{LUMO}}-E_{\mathrm{HOMO}}$$
(b) Hardness (η) is a measure of the resistance to changes in electron density:
$$\eta =E_{\mathrm{LUMO}}-E_{\mathrm{HOMO}}/2$$
(c) Softness (*s*) is the reciprocal of hardness:
s=1/η

### 2.7 Molecular Dynamics Simulations

The structural stability of the lead phytocompounds complexed with protein and EDTA was determined by using GROMACS-2019 and the Charmm36 all-atom force field to generate ligand topology. The systems were equilibrated under periodic boundary conditions with a cubic box of 1.0 nm and solvated with the TIP3P water model. Next, chlorine and sodium ions were added to neutralize the system, followed by energy minimization using the steepest descent and conjugate gradient algorithms for 50,000 steps. To equilibrate the system, NPT and NVT ensembles were utilized for maintaining a constant temperature of 300 K and constant pressure of 1 bar. Finally, the MDS was performed for a 100 ns time period for each system.^39^

The results were analyzed using tools integrated into the GROMACS package. The root mean square deviation (RMSD) shows structural stability, root mean square fluctuations (RMSF) show fluctuation, radius of gyration (*R*_g_), and hydrogen bonding were employed for system analysis. The graph was plotted using Xmgrace tool.^40^

## 3 Results

The potential of natural compounds, particularly antimicrobial agents, remains largely unexploited. Screening phytocompounds against specific biological targets combined with structural dynamics studies is a highly recommended strategy in traditional drug design for the development of novel and effective drug molecules.

### 3.1 Structural Validation of the Target Protein

NDM-1 remains the major target hindering the outcomes of TD and is a critical therapeutic target. The 3-dimensional structure of the β-lactamase receptor was acquired from the Protein Data Bank (PDB ID: 6TWT). It was determined by X-ray diffraction at a resolution of 0.95 Å and was visualized by the Schrodinger tool, and no mutations were observed in the structure. The sequence length of the protein was 243 amino acids. The Ramachandran plot from PROCHECK indicates that 91.6% of the residues fall within the most favored region, 7.9% in the additionally allowed region, and 0.5% in the disallowed region.

### 3.2 *In Silico* Pharmacokinetic and ADMET Analysis

The pharmacokinetic properties are among the most challenging aspects of designing a drug. These properties are crucial for determining whether a molecule is a viable drug candidate. They depend on several factors, including the Lipinski rule of five, as well as the ADME characteristics depicted in Table 1.

**Table 1:** *In Silico* Pharmacokinetic and ADME Analyses of Selected Compounds Using the QikProp Tool^a^

Pubchem ID	compound name	% HOA	QPlog Po/w	QPlogS	QPlog HERG	QPlog BB	RO5
1183	vanillin	82.06	1.004	–1.134	–3.376	–0.653	0
8468	vanillic acid	66.92	1.045	–1.395	–1.606	–0.881	0
444539	*trans*-cinnamic acid	79.52	1.910	–1.645	–2.393	–0.555	0
10742	syringic acid	66.05	0.971	–1.621	–1.612	–0.978	0
338	salicylic acid	76.70	2.259	–1.616	–1.615	–0.651	0
5280343	quercetin	52.19	0.385	–2.884	–5.075	–2.380	0
72	protocatechuic acid	52.90	0.031	–0.799	–1.515	–1.223	0
135	*p*-hydroxybenzoic acid	64.10	0.585	–1.537	–1.632	–0.789	0
637542	*p*-coumaric acid	67.47	1.439	–1.676	–2.280	–1.081	0
637540	*o*-coumaric acid	69.34	1.487	–1.654	–2.280	–0.995	0
5280863	kaempferol	64.12	1.061	–3.142	–5.178	–1.864	0
780	homogentisic acid	58.09	0.444	–0.971	–1.549	–1.164	0
5280373	biochanin A	90.84	2.562	–3.601	–5.119	–0.873	0
**5281672**	**myricetin**	**27.40**	**–0.281**	**–2.645**	**–4.973**	**–2.903**	**1**
**1794427**	**chlorogenic acid**	**16.49**	**–0.250**	**–2.520**	**–3.311**	**–3.317**	**1**
689043	caffeic Acid	54.36	0.557	–1.308	–2.181	–1.547	0
445858	ferulic acid	67.23	1.376	–1.871	–2.242	–1.177	0
439246	naringenin	74.458	1.653	–3.423	–4.964	–1.396	0
**10621**	**hesperidin**	**0**	**–1.349**	**–3.739**	**–6.398**	**–4.685**	**3**
9064	cianidanol	60.577	0.479	–2.614	–4.744	–1.881	0
7121	3,4-dimethoxybenzoic acid	82.587	2.155	–2.007	–1.552	–0.455	0
3469	2,5-dihydroxybenzoic acid	58.853	0.792	–1.060	–1.513	–1.147	0
Chelating Agent							
6049	EDTA	0	–3.374	0.920	2.311	–2.554	0

a The key parameters are summarized here, including lipophilicity (logP), aqueous solubility (logS), drug likeness, and absorption metrics.

Based on the protocol of the Qikprop module, molecules with human oral absorption ≥30% are considered drug candidates. The selected phytocompounds show human oral absorption >30%, except hesperidin, which violates the three rule of five, chlorogenic acid, myricetin, and chelating agent (EDTA), which cannot be considered a drug molecule.

### 3.3 Molecular Docking Studies

Toward the comprehensive search of phytocompounds, a total of 22 compounds were docked against the NDM-1 receptor through the Glide-XP mode in Schrodinger to analyze the binding affinity. Before docking the binding site was predicted by the Sitemap module in Schrodinger, and the binding site residues (93, 122, 123, 124, 154, 189, 207, 208, 210, 211, 212, 214, 215, 216, 217, 219, 220, 248, 249, 250, 251, 301, 302) were defined in the grid generation. The Glide gscore ranges from −9.107 to −5.076 kcal/mol. The molecular docking results of the compounds against the NDM-1 receptor are shown in Table 2.

**Table 2:** Docking Scores, Binding Energies, and Glide Energy for Lead Phytocompounds from C. anguria against NDM-1 (PDB ID: 6TWT)

protein	Pubchem ID	com. name	docking score (kcal/mol)	XPG score	glide score	glide energy
**(NDM-1)** PDB ID: 6TWT	780	homogentisic acid	–8.818	–8.818	–8.818	–46.498
	689043	caffeic acid	–8.663	–8.663	–8.663	–44.076
	72	protocatechuic acid	–8.121	–8.121	–8.121	–32.843
	5281672	myricetin	–8.020	–8.066	–8.066	–42.318
	637540	*o*-coumaric acid	–7.842	–7.844	–7.844	–24.610
	637542	*p*-coumaric acid	–7.839	–7.839	–7.839	–23.703
	3469	2,5-dihydroxybenzoic acid	–7.750	–7.750	–7.750	–24.732
	Chelating Agent					
	6049	EDTA	–5.178	–7.258	–7.258	–32.3

The three hit lead molecules are homogentisic acid, caffeic acid, and protocatechuic acid with Glide g-scores of −8.818, −8.663, and −8.121 kcal/mol, respectively. It formed hydrogen bonds with GLN 123 and ASN 220, and metal coordination with Zn^2+^ ions, as depicted in Figure 2.

**Figure 2:**
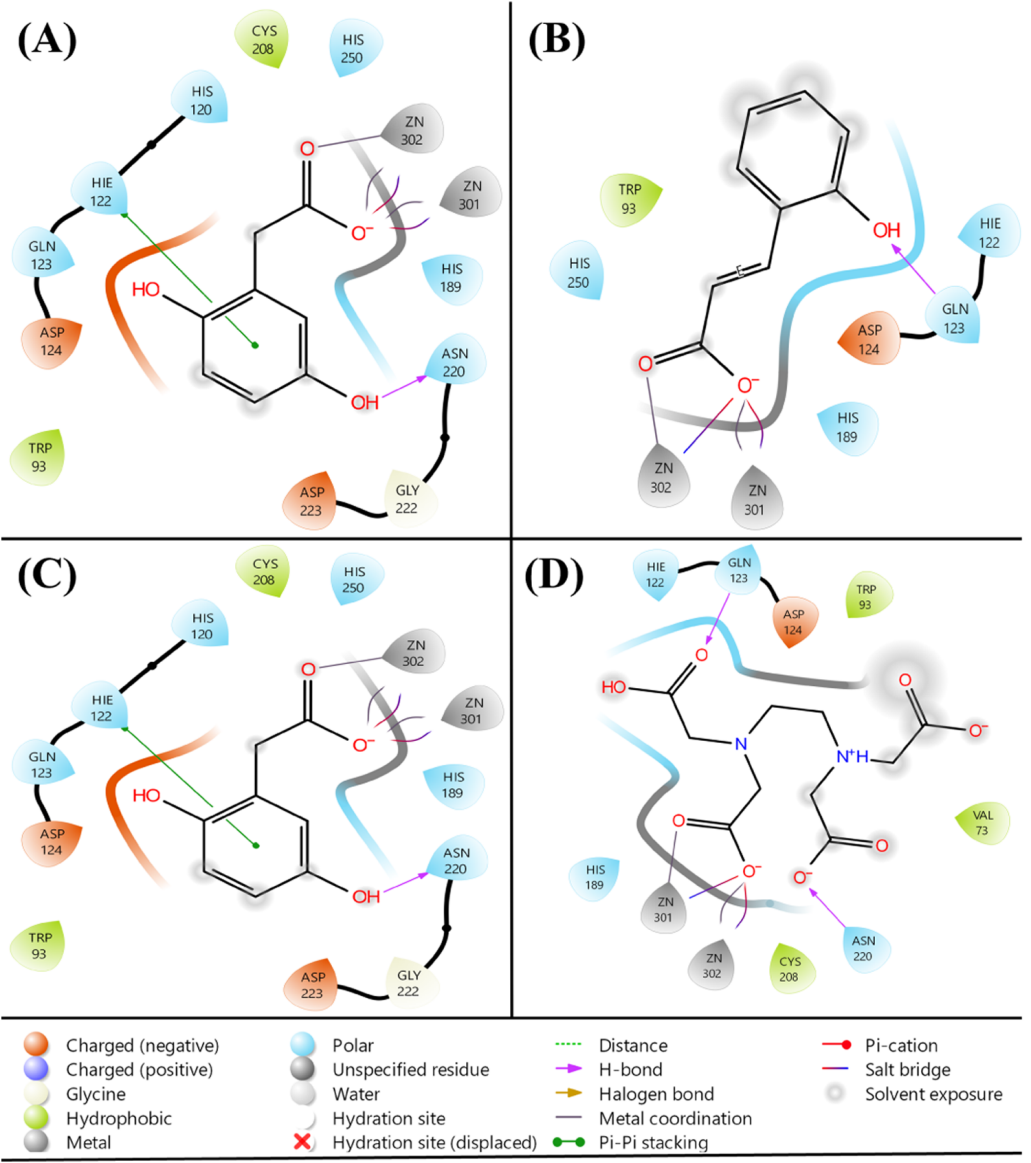
Molecular docking interactions of compounds from C. anguria and chelating compound EDTA with the active site of the NDM-1 protein. The interactions with 6TWT protein residues are shown for (A) homogentisic acid, (B) caffeic acid, (C) protocatechuic acid, and (D) EDTA.

Toxicity assessments for the lead compounds and the chelating agent were conducted using the ProTox-III web server, as shown in Table 3. The compounds protocatechuic acid (72) and EDTA demonstrated activity in carcinogenicity, which may lead to cancer.

**Table 3:** Predicted Toxicity Profiles of the Top Five Lead Compounds from C. anguria Using the ProTox-II Web Server, Including Various Toxicity End Points

	compounds			chelating agent
toxicity	homogentisic acid	caffeic acid	protocatechuic acid	EDTA
hepatotoxicity	inactive	inactive	inactive	inactive
neurotoxicity	inactive	inactive	inactive	inactive
cardiotoxicity	inactive	inactive	inactive	inactive
carcinogenicity	inactive	inactive	active	active
immunotoxicity	inactive	inactive	inactive	inactive
mutagenicity	inactive	inactive	inactive	inactive
cytotoxicity	inactive	inactive	inactive	inactive

### 3.4 Prime: MM-GBSA Analysis

The binding free energies of the selected hit molecules within the active site of NDM-1 were estimated by using MM/GBSA calculations in the protein–ligand complexes. This analysis reveals the binding energy of the top three hit molecules complexed with the NDM-1 receptor, i.e., homogentisic acid (−46.498 kcal/mol), caffeic acid (−48.076 kcal/mol), protocatechuic acid (−35.843 kcal/mol), and EDTA (−24.09 kcal/mol). This results in the strong binding affinity of the lead three molecules and EDTA, which supports the glide docking results.

### 3.5 DFT Calculation

The top three scoring molecule conformations were analyzed by using frontier molecular orbital analysis to predict the electrostatic properties, chemical stability, and reactivity. Electrostatic potential maps were generated to identify regions of high and low electron density within the molecule. The HOMO, LUMO, and MESP regions of the compounds are depicted in Figure 3, in which the red color shows the negative phase and the blue color shows the positive phase in the HOMO and LUMO plots. The energy gap of lead molecules was in the range of 0.1480 to 0.4148 eV, as shown in Table 4 with its electronegativity, hardness, and softness.

**Figure 3:**
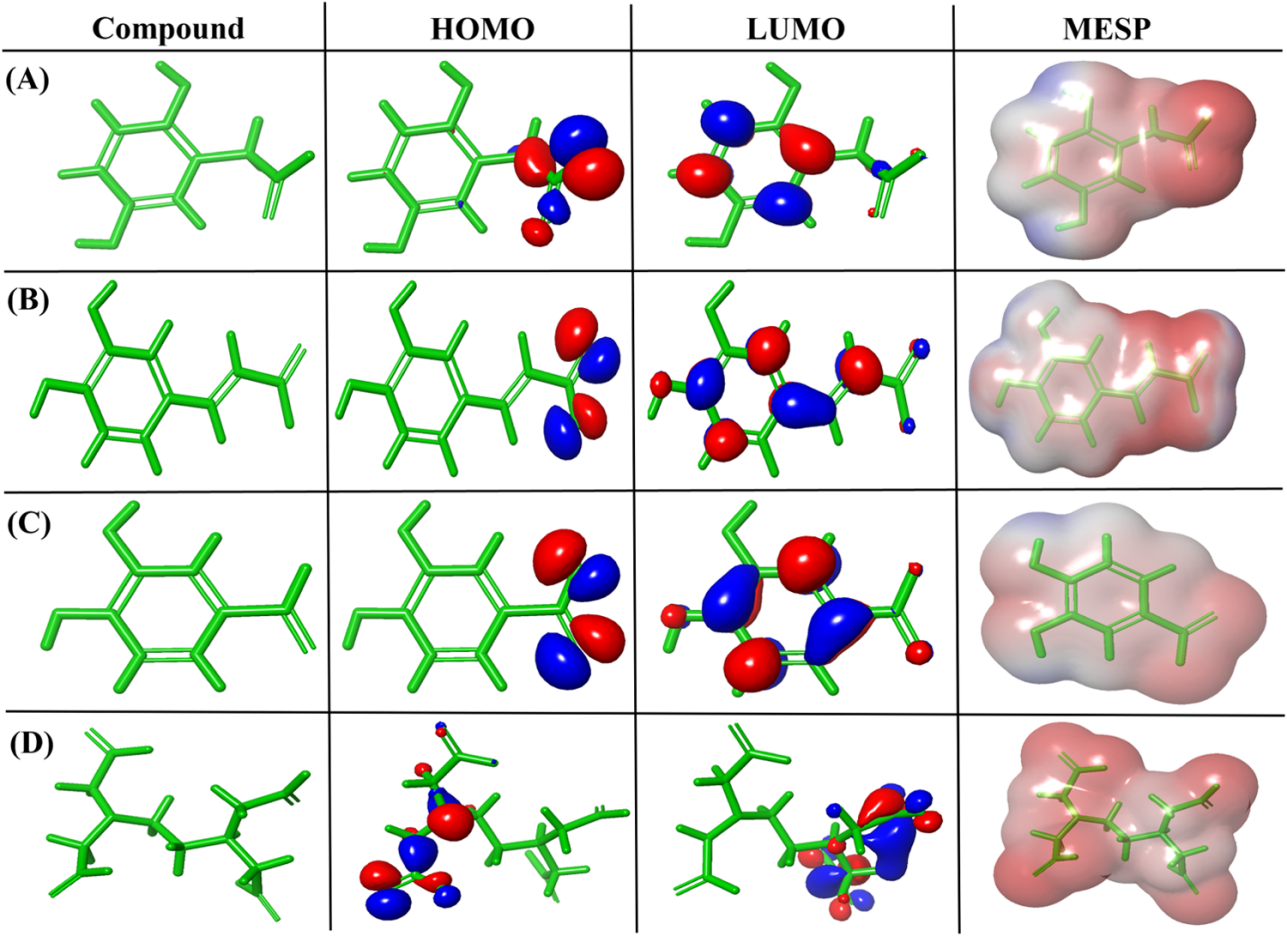
HOMO, LUMO and MESP regions of the phytocompounds from C. anguria. (A) homogentisic acid, (B) caffeic acid, (C) protocatechuic acid, and (D) chelating agent EDTA.

**Table 4:** HOMO and LUMO Energies of the Phytocompounds Homogentisic Acid, Caffeic Acid, Protocatechuic Acid, and Chelating Agent EDTA

s. no.	compound	HOMO (eV)	LUMO (eV)	energy gap (eV)	hardness (eV)	softness (eV)
1	homogentisic acid	–0.0201	0.1280	0.1481	0.07	13.51
2	caffeic acid	–0.2314	0.0936	0.3247	0.16	6.17
3	protocatechuic acid	–0.0273	0.1418	0.4148	0.20	4.83
4	EDTA	0.1433	0.380	0.5233	0.26	3.84

Hardness (η) and softness (*s*) are key concepts in DFT that describe a molecule’s resistance or susceptibility to changes in its electron density. Molecules with low hardness and high softness are highly reactive in nature.

### 3.6 Molecular Dynamics (MD) Simulation

MD simulation was performed to study the conformational stability of the protein and ligands. Variation at the atomistic level of the protein–ligand and its complexes in the dynamic environment was determined by using MD simulation.^41^ In the present study, the parameters such as RMSD, RMSF, *R*_g_, and hydrogen bond formation for the apo and complexes were evaluated over a 100 ns simulation.

#### 3.6.1 RMSD

RMSD values were calculated for the backbone atoms of the protein and each complex relative to their respective initial structures. A low RMSD value indicates that the protein maintains a similar structure, suggesting stability throughout the simulation, and vice versa. The dynamic variation in the backbone of the three lead complexes was determined by monitoring the RMSD during the molecular simulation. The plot of the complex reveals that RMSD ranges between 0.1 and 0.3 nm, and the average is noticed at 0.15 nm. It was found to be very consistent, and no significant deviations are observed in Figure 4. RMSD plots indicate that the binding of inhibitors does not induce any greater conformational changes in the backbone of the NDM-1 receptor.

**Figure 4:**
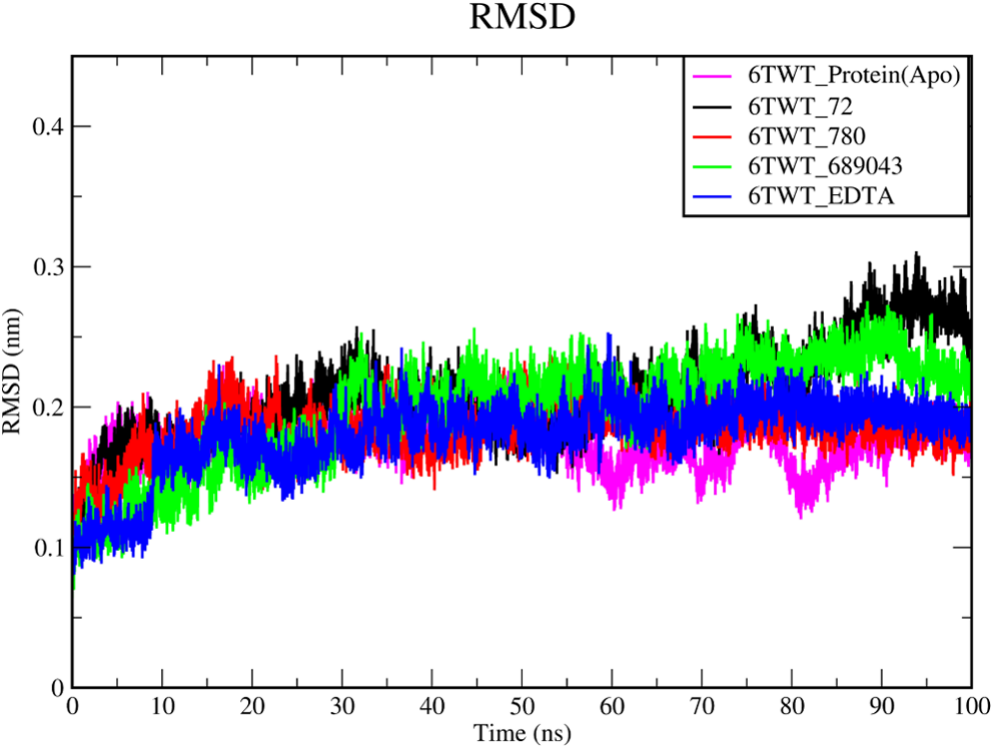
RMSD of the protein backbone atoms during the 100 ns MD simulation. The deviations in apo 6TWT_Protein­(Apo) (magenta) and complexes 6TWT_72 (protocatechuic acid), 6TWT_780 (homogentisic acid), 6TWT_689043 (caffeic acid), and 6TWT_EDTA are represented by the black, red, green, and blue lines, respectively.

#### 3.6.2 RMSF

The RMSF graph illustrates the fluctuation of residues within the protein throughout the molecular dynamics simulation. In this graph, the *x*-axis represents the residue index, while the *y*-axis indicates the RMSF value in nanometers (nm). Regions exhibiting higher RMSF values correspond to residues with greater flexibility, often indicating the protein’s loop regions or terminal ends. Fluctuations occur in the residues between 63 and 75, 120–125, 144–156, 169–177, and 213–229 in the loop regions, as shown in Figure 5.

**Figure 5:**
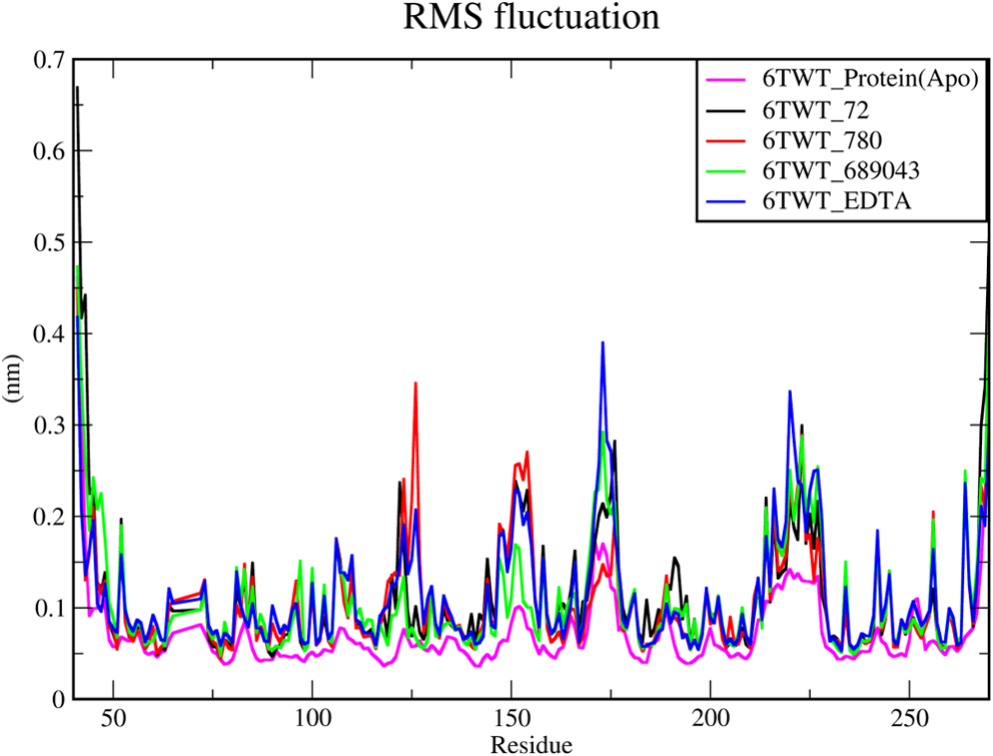
RMS fluctuation analysis of protein–ligand complexes over a 100 ns MD simulation. The fluctuation of the apo 6TWT_Protein­(Apo) (magenta) and complexes 6TWT_72 (protocatechuic acid), 6TWT_780 (homogentisic acid), 6TWT_689043 (caffeic acid), and 6TWT_EDTA is represented by the black, red, green, and blue lines, respectively.

#### 3.6.3 Radius of Gyration

The graph representing *R*_g_ provides insights into a molecular system’s overall compactness or expansion under varying conditions. It measures the mass distribution around the center of mass over a 100 ns time interval of the molecules, indicating their structural conformation. The low *R*_g_ values indicate that the structure of proteins is more compact and atoms are closer to the center of mass, while higher values indicate that the atoms are spread further from the center. The gyration values range between 1.65 nm and 1.7 nm, suggesting that the structure of the molecule is more compact, as shown in Figure 6.

**Figure 6:**
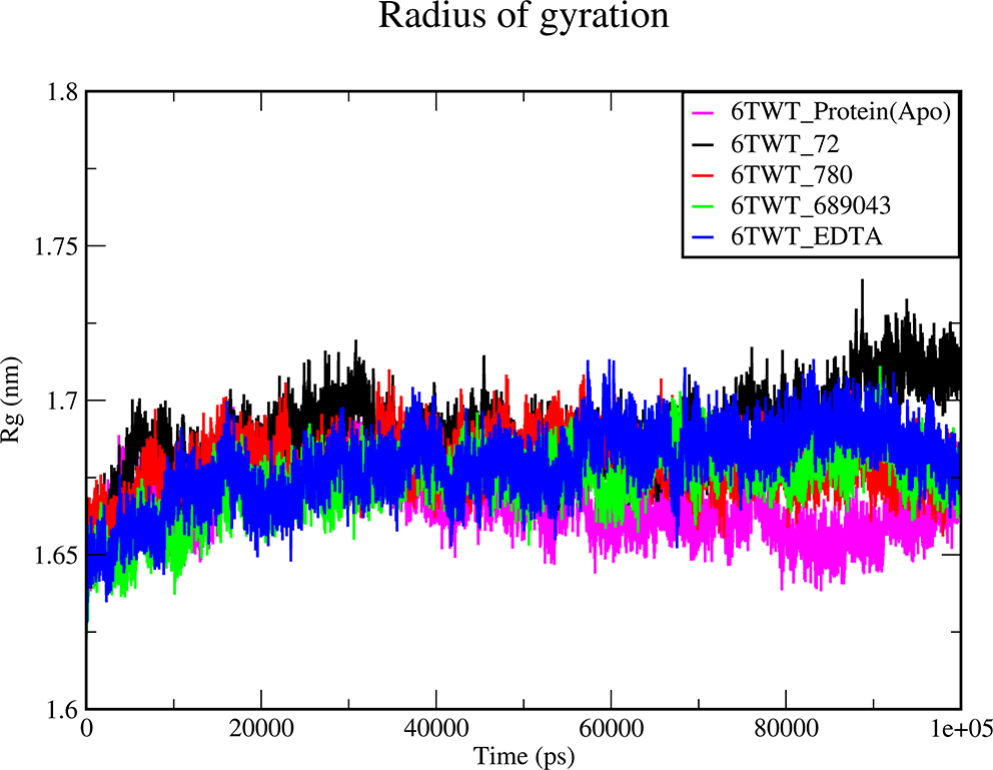
The plot shows the variation in the radius of gyration, highlights the structural transitions, and shows the overall conformation of the complexes. 6TWT_Protein­(Apo), 6TWT_72 (protocatechuic acid), 6TWT_780 (homogentisic acid), 6TWT_689043 (caffeic acid), and 6TWT_EDTA are represented by the magenta, black, red, green, and blue lines, respectively.

#### 3.6.4 Hydrogen Bond Analysis

Hydrogen bond analysis provides insight into the structural stability and compactness of the protein and complex during the simulation period, as depicted in Figure 7. In the intramolecular hydrogen bond analysis, a consistent number of hydrogen bonds formed throughout the trajectory suggested that the native protein maintained its native secondary and tertiary structures under simulated conditions. In the present study, the number of intramolecular hydrogen bonds in both the apo form and ligand-bound complexes remained relatively stable during the 100 ns simulation (Figure 7A), indicating no major structural distraction. This suggests that ligand binding did not adversely affect the protein’s internal hydrogen bonding network, supporting the structural integrity and reliability of the protein–ligand complexes throughout the simulation period. The phytocompounds complexed with the receptor revealed that 2–4 hydrogen bonds and EDTA revealed that 1–2 bonds were formed throughout the simulation (Figure 7B). These results showed that the screened phytocompounds maintained a good interaction with the active site and suggested that the docking complexes were stable throughout the 100 ns simulation.

**Figure 7:**
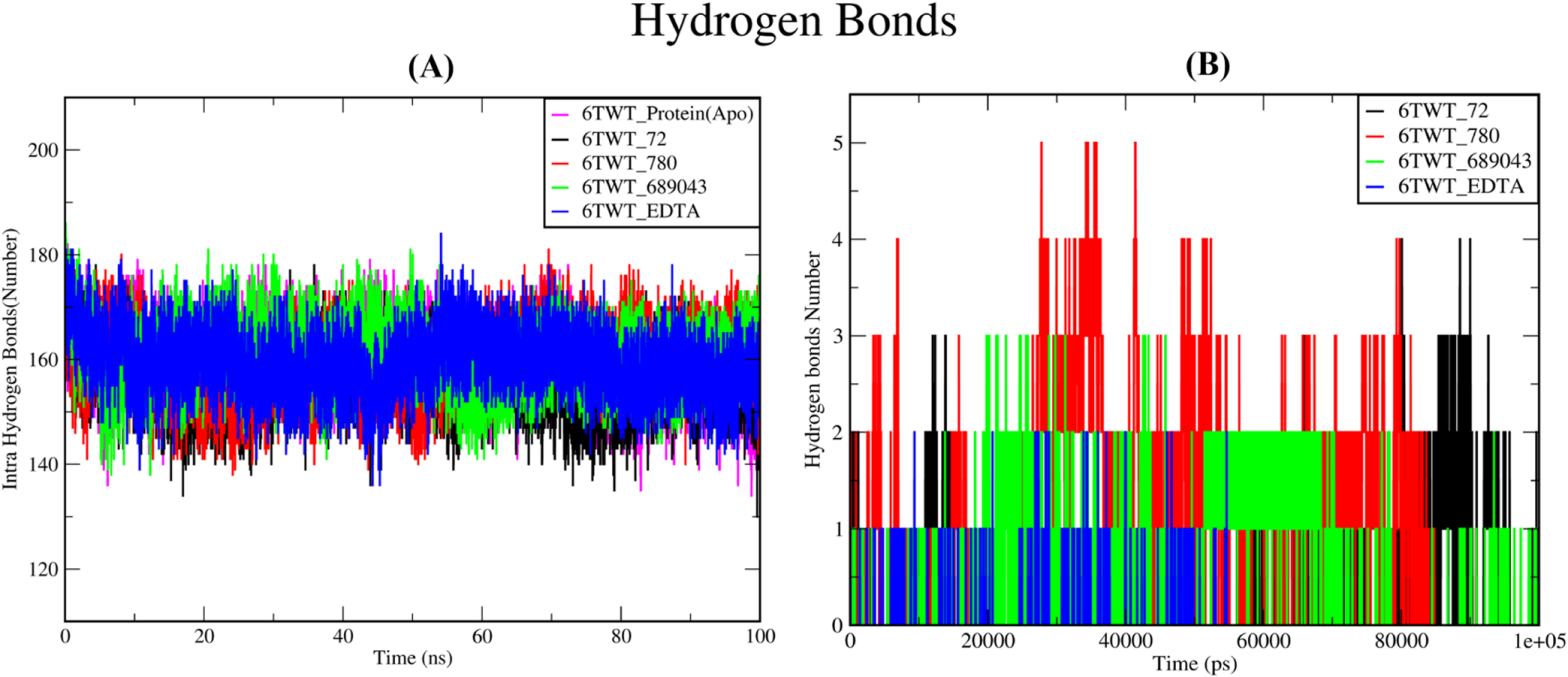
Hydrogen bond formation throughout the MD simulation. (A) Intramolecular hydrogen bonds. (B) Hydrogen bonds between the complexes 6TWT_72 (protocatechuic acid), 6TWT_780 (homogentisic acid), 6TWT_689043 (caffeic acid), and 6TWT_EDTA are represented by the black, red, green, and blue lines, respectively.

## 4 Discussion

The emergence of NDM-1 poses a significant global health threat by conferring resistance to β-lactam antibiotics, including carbapenems, which are often considered the last line of defense against multidrug-resistant bacterial infections.^42^ Standard antibiotic treatments for TD are still ineffective, necessitating the use of more potent and expensive antibiotics.^43^

E. coli and K. pneumoniae are responsible for many nosocomial infections in immunocompromised patients, and these organisms carry the NDM-1 gene responsible for TD.^44^ The infections caused by bacteria that produce NDM-1 are associated with longer duration of illness, increased risk of medical complications, and higher healthcare costs due to the need for more intensive treatment and prolonged hospital stays; in some cases, it leads to death.^45^ These bacteria, equipped with NDM-1, are a critical target in the treatment of antibiotic-resistant infections due to their ability to hydrolyze and inactivate a broad spectrum of β-lactam antibiotics, including penicillins, cephalosporins, and carbapenems, which are often used as last-resort treatments.^46^

NDM-1 is particularly challenging because of the lack of clinically approved inhibitors capable of neutralizing its zinc-dependent enzymatic activity, as traditional β-lactamase inhibitors are ineffective against metallo-β-lactamases.^47^ Targeting NDM-1 offers a promising way to restore the efficacy of β-lactam antibiotics by developing novel inhibitors, such as zinc-chelating agents or structure-based small molecules designed to block its active site.^20^ Therefore, addressing NDM-1 through innovative drug discovery approaches is vital for managing and mitigating the growing threat of antimicrobial resistance.

The molecules that chelate the characteristics of Zn^+^ ions in the NDM-1 gene act as an inhibitor^48^ and inhibit the enzyme’s ability to hydrolyze β-lactam antibiotics.^49^ Few compounds may act as viable inhibitors that bind to the active site of NDM-1 and prevent the substrate from binding to antibiotics, leading to resistance.^14^ Bioactive compounds from medicinal plants may lead to synergistic effects, enhancing the overall inhibitory activity against NDM-1.^50,51^ Many compounds in C. anguria have metal-binding properties that chelate zinc ions in the active site of NDM-1, essential for its enzymatic activity.

Molecular docking results revealed that several phytocompounds, chlorogenic acid, hesperidin, homogentisic acid, caffeic acid, and protocatechuic acid from C. anguria exhibited strong binding affinities toward the active site of NDM-1, comparable to the metal chelator EDTA. Among them, chlorogenic acid and hesperidin did not meet ADME properties, and thus cannot be considered drug molecules. In addition, EDTA showed 0% drug-likeness and is immunotoxic. Notably, compounds such as homogentisic acid, caffeic acid, and protocatechuic acid demonstrated significant interactions with key catalytic residues GLN 123, ASN 220, and Zn^2+^ ions, which are essential for enzymatic activity. These interactions suggest that the identified compounds can effectively chelate zinc cofactors, which are crucial for the hydrolysis of β-lactam antibiotics. The compounds taniborbactam and xeruborbactam are currently in clinical trials targeting both MBLs and SBLs, and further clinical research is essential to confirm their efficacy. Additionally, ANT2681 is a promising molecule specifically designed to inhibit MBLs and is advancing toward clinical development.^52^ 4-Amino-1,2,4-triazole-3-thione derivatives, 3-aryl-6,7-dihydro-[1,2,4]­triazolo­[3,4-*b*]­[1,3]­thiazines, 3-phenyl-5,6-dihydro-[1,2,4]­triazolo­[3,4-*b*]­[1,3] thiazoles, 1-substituted-1H-imidazole-2-carboxylic acid derivatives,^53^ Indole-2-carboxylic acid derivatives, 3,4-dihydroxyphenylacetic acid derivatives are the zinc inhibitors, evaluated for inhibitory activity against five representative MBLs (L1, VIM-4, VIM-2, NDM-1, and IMP-1), all compounds exhibited activity against at least one enzyme, with the most promising inhibitor demonstrating significant potential against MBLs.^54^ β-Lactam conjugates with pyridyl-containing chelators, tormentic acid, and combined pyridyl- and carboxylate-containing chelators, β-lactam conjugates with carboxylate-containing chelators, carboxylate-containing chelators, and 2,6-dipicolinic acid^55^ are the metal chelators and its toxicity experiments demonstrated that a carboxylic derivative exhibited low *in vitro* cytotoxicity and minimal *in vivo* toxicity. Additionally, *in vivo* studies revealed that the combined treatment of this derivative with meropenem significantly improved the survival probability of mice infected with MBL-producing K. pneumoniae.^55^ Prime MM-GBSA is a widely used postdocking method for calculating the binding free energies. The phytocompounds show better binding free energies when compared to EDTA.

The stability of lead phytocompounds and NDM-1 complexes was assessed further through MD simulations. Deviation, fluctuation, and radius of gyration analyses indicated that the protein–ligand and EDTA complexes remained stable during the simulation, with only minor fluctuations. Furthermore, hydrogen bond analysis confirmed the persistence of interactions over time, reinforcing the potential inhibitory activity of the selected phytocompounds. There is some disruption in the hydrogen bonding interactions between EDTA and the receptor during the simulation, indicating that the hydrogen bonds do not remain consistently stable throughout the trajectory. This fluctuation suggests that dynamic interactions are potentially influenced by conformational changes in the receptor–ligand complex or solvent effects.

Natural inhibitors offer several potential advantages over synthetic compounds in combating NDM-1-mediated antibiotic resistance.^56^ The compounds derived from various plant sources possess complex and unique chemical scaffolds that may enhance their binding specificity and affinity for biological targets like NDM-1 enzyme with lower toxicity and better biocompatibility, reducing the risk of adverse effects that are commonly seen with synthetic drugs.^57^ Naturally derived inhibitors may exhibit multitarget activity, helping to suppress multiple resistance mechanisms simultaneously, which can reduce the chances of bacteria developing resistance. Their abundance and ecofriendly nature also make them attractive compounds for sustainable drug discovery. These characteristics support the potential use of natural compounds as promising leads for the development of safer and more effective NDM-1 inhibitors.

This study highlights the potential of C. anguria bioactive compounds as natural inhibitors of NDM-1. However, although computational predictions provide valuable insights, experimental validation through enzyme inhibition assays, bacterial susceptibility tests, and toxicity profiling of phytocompounds is essential to confirm their efficacy as a potential drug candidates. Future studies should also explore structural modifications to enhance potency and pharmacokinetic properties for potential drug development.

## 5 Conclusions

NDM-1 is critical for developing effective inhibitors to combat antibiotic-resistant bacterial infections. The selective compounds from C. anguria are a natural source for developing NDM-1 inhibitors for treating TD by chelating the zinc ions in the NDM-1 enzyme. By targeting the metalloenzyme’s active site through metal chelation, which acts as a competitive inhibition mechanism, these compounds could potentially restore the effectiveness of β-lactam antibiotics against resistant bacterial strains. A promising hypothesis arising from this study is that phytocompounds from *C. anguria* can restore the effectiveness of β-lactam antibiotics by competitively binding to the zinc-dependent active site of NDM-1, thereby disrupting its hydrolytic activity. This study suggests that phytocompounds derived from C. anguria exhibit potential therapeutic effects against TD, particularly by targeting the NDM-1 enzyme associated with antibiotic resistance. Comprehensive *in vitro* and *in vivo* experiments are essential to evaluate the efficacy of these compounds in inhibiting NDM-1 activity. Such validation will help researchers to develop effective treatments for TD and other infections caused by NDM-1-producing pathogens. This holistic approach validates traditional remedies and fosters the modernization and globalization of plant-based bioactive molecules in contemporary medicine.
